# Clinicopathologic features of de novo non-alcoholic steatohepatitis in the post-transplant setting

**DOI:** 10.1186/s13000-022-01247-y

**Published:** 2022-08-10

**Authors:** Dana Balitzer, Jia-Huei Tsai, Ryan M. Gill

**Affiliations:** 1 Department of Pathology, San Francisco VA Health Care System, San Francisco, CA USA; 2 Department of Pathology, University of California, San Francisco, CA USA; 3 Department of Pathology, National Taiwan University Hospital, Taipei, Taiwan; 4 Graduate Institute of Pathology, College of Medicine, National Taiwan University, Taipei, Taiwan

**Keywords:** Non-alcoholic steatohepatitis (NASH), Hepatitis C Virus, Liver transplantation, Allograft, Ballooned hepatocyte

## Abstract

**Background:**

Non-alcoholic steatohepatitis (NASH) has become an increasingly recognized problem in patients after orthotopic liver transplant. The aims of this study were to compare the clinicopathologic features of recurrent and de novo NASH.

**Methods:**

From 1995 to 2016, we performed a retrospective review of patients with a histological diagnosis of non-alcoholic steatohepatitis made more than 6 months after liver transplant at University of California, San Francisco. The cases were categorized into de novo (*n* = 19) or recurrent steatohepatitis (*n* = 37).

**Results:**

Hepatitis C virus (HCV) infection-related cirrhosis was the most common etiology of transplantation in de novo NASH (78% of cases, *n* = 29). There was no difference in glycogenosis or presence of grade 3 steatosis. More recurrent NASH biopsies had small ballooned hepatocytes (62.5% of cases) compared to de novo NASH (26.7%) (*p* = 0.03), and were less likely to show prominent portal inflammation (5% versus 40.5%, *p* = 0.0049). The diagnosis of recurrent NASH was made significantly sooner after transplantation than the diagnosis of de novo NASH (2.8 years versus 4.8 years, *p* = 0.02).

**Conclusions:**

Overall, our results support that recurrent NASH demonstrates distinct clinicopathologic features compared to de novo NASH arising in the post-transplant setting.

## Background

Non-alcoholic fatty liver (NAFL) and non-alcoholic steatohepatitis (NASH) are two histologic diagnoses that comprise the histologic spectrum of disease referred to as non-alcoholic fatty liver disease (NAFLD); however, only NASH harbors a significant risk of progressive liver fibrosis and eventual cirrhosis [1]. Well-established risk factors for the development of NAFLD and NASH include obesity, metabolic syndrome, hyperlipidemia, and type 2 diabetes mellitus (DM) [2]. Due to the increasing prevalence of obesity and type 2 diabetes mellitus, NAFLD has become an increasingly prevalent disease around the world and NASH is now the second leading cause of liver transplantation in the United States [3, 4].

Although liver transplantation successfully treats NASH-related liver cirrhosis, both recurrent and de novo NAFLD have become an increasingly recognized problem in patients after orthotopic liver transplant [5]. Risk factors for the development of fatty liver disease in the posttransplant setting are similar to risk factors in the pre-transplant setting, including obesity, hypertension, and diabetes [6–8]. One study of 248 patients undergoing liver transplant demonstrated a significant increase in the prevalence of metabolic syndrome in the posttransplant period, up to 51.9%, compared to 5.4% in the pretransplant period [9], which is likely to also contribute to post-transplant NASH prevalence. Patients in the post-transplant setting also have unique risk factors, including the use of immunosuppressant drugs such as steroids and calcineurin inhibitors [10].

In patients with a prior diagnosis of NASH, NAFLD frequently recurs in the first 5 years after transplantation [11]. A recent study of 103 patients who underwent liver transplantation for NASH demonstrated a high frequency of recurrent NAFLD in 88.2% of patients [12]. Histologically, recurrent post-liver transplant NAFLD is usually a mild disease, with only a minority of cases (less than 10%) demonstrating ballooned hepatocytes or perisinusoidal fibrosis diagnostic of NASH [7].

The reported incidence of recurrent NASH is less common, ranging from 4% to 41.2% [6, 12–17]. Steatosis appears to be a prerequisite for the development of recurrent NASH [6], but the clinicopathologic features have not been well-characterized. There have been fewer studies evaluating de novo NASH in the posttransplant setting, with a reported incidence ranging from 9–13% [18, 19]; the clinicopathologic findings of de novo NASH have also not been well-characterized. The aim of this study was to evaluate clinicopathologic features and compare the differences between recurrent and de novo NASH.

## Methods

### Patient selection

From 1995 to 2016, a retrospective review of patients with a histological diagnosis of steatohepatitis made more than 6 months after liver transplant at University of California, San Francisco was performed. The study was approval by the Committee on Human Research, the Department of Pathology, University of California, San Francisco (San Francisco, CA). Cases were excluded if there was documented alcohol abuse or clinically suspected drug-related steatohepatitis. The clinical data, including demographics, clinical information, medical conditions, body mass index (BMI), and long-term outcome, were obtained from medical records. All the post-transplant biopsy reports of recurrent diseases, fibrosis staging or rejection (i.e. acute cellular rejection or chronic rejection) were recorded. All patients were designated as recurrent or de novo NASH. The recurrent NASH group was defined as patients who received liver transplant for a clinicopathologic diagnosis of NASH-related cirrhosis or cryptogenic cirrhosis with one or more metabolic comorbidities (i.e. BMI > 30 or a clinically established diagnosis of pre-transplant hypertension, hyperlipidemia or diabetes mellitus type 2). The de novo NASH group consisted of patients whose transplant indication was a liver disease other than NASH.

### Histological examination

All biopsies with a diagnosis of steatohepatitis were evaluated by DJB, JHT and RMG to confirm the diagnosis of NASH by using established histologic criteria of lobular inflammation, steatosis (> 5%), and the presence of ballooned hepatocytes [20–23]. Additional pathologic features characterized include steatosis (grade, scale 0–3 [20–22], portal inflammation (which was subdivided into two categories, mild or “more than mild” [24]), presence of any centrizonal arteries [25], and glycogenosis (present or absent [23]). The biopsies were assessed for the presence of ballooned hepatocytes [20–22] which were further characterized morphologically by size as large (i.e. ≥ 1.5 × adjacent hepatocyte size) or small (< 1.5 × adjacent hepatocyte size) Trichrome stain was used to stage fibrosis using the Brunt-Kleiner method [20–22]. The presence of pericellular fibrosis and portal fibrosis was also assessed.

### Clinical evaluation

The patients' medical records were reviewed for history of liver-related problems, duration of liver disease, viral serology, viral genotypes and viral load, autoimmune markers, BMI, clinical diagnosis of metabolic syndrome or related conditions (i.e. hypertension, hyperlipidemia and diabetes mellitus type 2) and any documented use of alcohol or other drugs. Posttransplant clinical course, complications, immunosuppressive regimen, and outcome were also determined.

### Clinicopathologic correlation

Based on the combination of the patient’s clinical presentation and characteristics, native liver and explant pathology, the patients were assigned to diagnostic categories.

### Statistical analysis

Comparisons of categorical variables were performed using the Pearson method or Fisher exact test when appropriate. Statistical calculation was applied using Student’s t test for continuous variables. Log-rank (Mantel-Cox) test was used to compare the survival distribution. All statistical results were considered significant if the *P* value was < 0.05.

## Results

### Clinical features

In our institute series, a total of 294 patients had post-transplant biopsies with steatosis.

A total of 56 patients were diagnosed with post-transplant NASH by pathologic evaluation. 37 patients were classified as de novo NASH and 19 patients were classified as recurrent NASH. Among post-transplant patients with steatosis, there was a 12.6% incidence of de novo NASH and 6.5% incidence of recurrent NASH. The indications for liver transplant in the two groups is summarized in Table 1.

**Table 1:** Pre-transplant Characteristics of de novo NASH

Cause	Number of patients	Percentage of Cases
**Hepatitis C Virus**	29	78.4%
**Hepatitis B virus**	2	5.4%
**Other**	Sarcoma (*n* = 1)	16.2%
	PBC (*n* = 1)	
	Oxalosis (*n* = 1)	
	Drug reaction (*n* = 1)	
	Alcoholic liver disease (*n* = 2)	

Hepatitis C virus (HCV) infection-related cirrhosis was the most common etiology of transplantation in de novo NASH and accounted for 78% (*n* = 29) of the indications. Of the cases of HCV, genotype 1a was the most frequent genotype (*n* = 9, 31.0%), followed by genotypes 3 (*n* = 7, 24.1%), 1b (*n* = 4, 13.8%), and 6 (*n* = 1,3.4%) and 2b (*n* = 1, 3.4%); genotypes were not available for seven patients (24.1%).

Of the cases transplanted for hepatitis C, 86% (*n* = 25) had histologic evidence of recurrent hepatitis C prior to or concurrent with the diagnosis of de novo NASH, while 70% of the cases transplanted for HCV (*n* = 20) had positive post-transplant hepatitis C viral loads. Three of the cases were treated as recurrent hepatitis C based on the histologic findings, without a documented recurrence by viral load testing. Two cases demonstrated non-specific histologic findings of active hepatitis and, while HCV was clinically excluded by a negative viral load, other de novo viral infections or autoimmune hepatitis remained considerations.

The clinical characteristics of the two groups are summarized in Table 2. The age (average of 62.7 years for recurrent NASH and 60.6 years for de novo NASH), sex distribution (68% and 62% male, respectively) and post-transplant BMI (34.2 and 36.1, *p* = 0.58) were similar between recurrent and de novo NASH. There were 16 (84%) recurrent NASH patients with a clinically established diagnosis of diabetes mellitus type 2, with 11 (58%) diagnosed prior to transplant and 5 (26%) diagnosed after transplantation. There were 21 (57%) de novo NASH patients with a clinical diagnosis of diabetes mellitus type 2, 17 of which were diagnosed with diabetes after transplantation (81%). Pre-transplant diagnosis of hyperlipidemia was also more common in patients with recurrent NASH (*n* = 4, 21%) compared to de novo NASH (*n* = 1, 3%, *p* = 0.0406). Post-transplant hyperlipidemia was similar between the two groups, in 37% (*n* = 7) of patients with recurrent NASH compared to 38% (*n* = 14) of de novo NASH (*p* > 0.9999).

**Table 2:** Clinical features of recurrent versus de novo NASH

Clinical Features	Recurrent NASH (*n* = 19)	De novo (*n* = 37)	*p* values
**Age (years)**	62.7 years	60.6 years	*p* = 0.378
**Gender (% male)**	68% (13)	62% (23)	*p* = 0.7715
**Hypertension (%)**			
Pre-transplant onset	21% (4/19)	54% (20/37)	*p* = 0.0238
Post-transplant onset	44% (8/19)	27% (12/37)	*p* = 0.3653
**Diabetes (%)**			
Pre-transplant onset	58% (11/19)	11% (4/37)	*p* = 0.0003
Post-transplant onset	26% (5/19)	46% (17/37)	*p* = 0.248
**Hyperlipidemia (%)**			
Pre-transplant onset	21% (4/19)	3% (1/37)	*P* = 0.0406
Post-transplant	37% (7/19)	38% (14/37)	*P* > 0.9999
**BMI (average, kg/m**^**2**^**)**	34.2	36.1	*p* = 0.58
**Time to diagnosis after transplant (years)**	2.8 years	4.8 years	*p* = 0.02

**Fig. 1:**
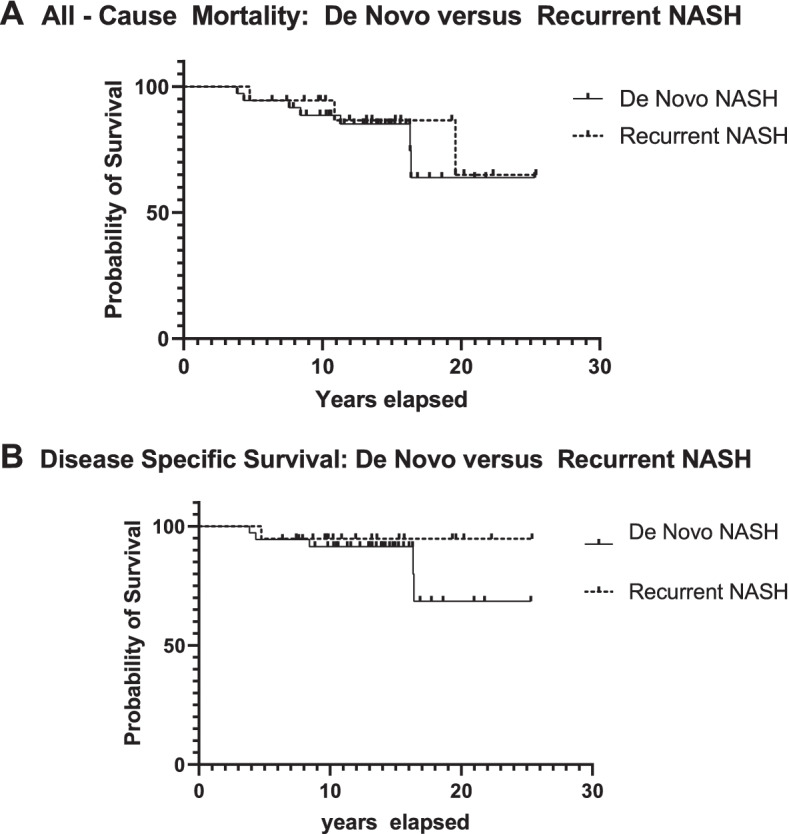
**A** Survival curves with all cause mortality in patients with de novo versus recurrent non-alcoholic steatohepatitis (NASH). There is no difference in all cause mortality by Log-rank (Mantel-Cox) test (*p* = 0.6479).** B** Survival curves with disease specific mortality in patients with de novo versus recurrent non-alcoholic steatohepatitis (NASH). There is no difference in disease specific mortality by Log-rank (Mantel-Cox) test (*p* = 0.3047)

The immunosuppressant medications are summarized in Table 3. There was no significant differences in the categories of maintenance immunosuppressant medications used between the two groups. We also evaluated the time period between liver transplantation and a post-transplant liver biopsy resulting in a pathologic diagnosis of non-alcoholic steatohepatitis. De novo NASH was diagnosed on average 4.8 years after transplant, which was significantly longer than the time between transplantation and a pathologic diagnosis of recurrent NASH (2.8 years, *p* = 0.02). There was no difference in all cause (Fig. 1A, *p* = 0.6479) or disease specific mortality, such as deaths secondary to metastatic hepatocellular carcinoma or liver failure (Fig. 1B, *p* = 0.3047), between patients with a diagnosis of de novo versus recurrent NASH.

**Table 3:** Maintenance Immunosuppression Therapy in de novo and Recurrent NASH

Immunosuppressant (± Mycophenolate)	De Novo NASH (*n* = 37)	Recurrent NASH (*n* = 19)	*P* value
Tacrolimus	21 (56.8%)	14 (73.7%)	0.2559
Cyclosporine	4 (10.8%)	2 (10.5%)	> 0.9999
Everolimus	1 (2.7%)	0 (0%)	> 0.9999
Sirolimus	2 (5.4%)	0 (0%)	0.5435
Mycophenolate only	6 (16.2%)	3 (15.8%)	> 0.9999

### Pathologic features

Histology from the post-transplant biopsies were reviewed on all patients. There was no difference in the proportion of patients diagnosed with acute cellular rejection in the two groups (42.1% in recurrent NASH vs. 18.9% in de novo NASH respectively, *p* = 0.1091).

The pathologic characteristics of the two groups are summarized in Table 4. Patients in both de novo NASH group (*n* = 23. 62.2%) and recurrent NASH group (*n* = 8, 42.1%) had steatosis on post-transplant biopsies prior to a diagnostic biopsy with steatohepatitis. In the de novo NASH group, *n* = 11 (28.9%) were initially diagnosed with steatosis after protocol biopsy for HCV surveillance, while the remainder were biopsied after presenting with elevated liver function tests. A subset of the recurrent NASH patients (*n* = 2, 10.5%) were also diagnosed as part of a protocol/surveillance biopsy. The remaining patients presented with elevated liver function tests.

**Table 4:** Pathologic Characteristics of Recurrent versus De Novo NASH

	**Recurrent NASH (*n* = 19)**	**De Novo NASH (*n* = 37)**	***P* values**
**Focal and/or diffuse glycogenosis**	26.3% (5/19)	43.2% (16/37)	*p* = 0.2559
**Ballooned hepatocytes**			
Any ballooned hepatocytes	84.2% (16/19)	81.1% (30/37)	> 0.9999
Small vs. large ballooned hepatocytes	62.5% (10/16)	26.7% (8/30)	*p* = 0.0271
Large ballooned hepatocytes (of total cases)	32.6% (6/19)	59.5% (22/37)	*p* = 0.0891
Small ballooned hepatocytes (of total cases)	52.6% (10/19)	21.6% (8/37)	*p* = 0.0329
**Severe steatosis (grade 3)**	42.1% (8/19)	18.9% (7/37)	*p* = 0.1091
**“More than mild” portal inflammation**	5% (1/19)	40.5% (15/37)	*p* = 0.0049
**Spotty necrosis**	42.1% (8/19)	54.1% (20/37)	*P* = 0.5731
**Mild lobular inflammation (lymphocytic)**	42.1% (8/19)	56.8% (21/37)	*P* = 0.3991
**Advanced fibrosis at diagnostic biopsy**	10.5% (2/19)	21.6% (8/37)	*P* = 0.4668
**Pericellular fibrosis**	47.4% (9/19)	37.8% (14/37)	*P* = 0.5717
**Portal fibrosis at diagnostic biopsy**	47.4% (9/19)	56.8% (21/37)	*P* = 0.578
**Centrizonal arterialization**	0% (0/19)	5.4% (2/37)	*P* = 0.5435

**Fig. 2:**
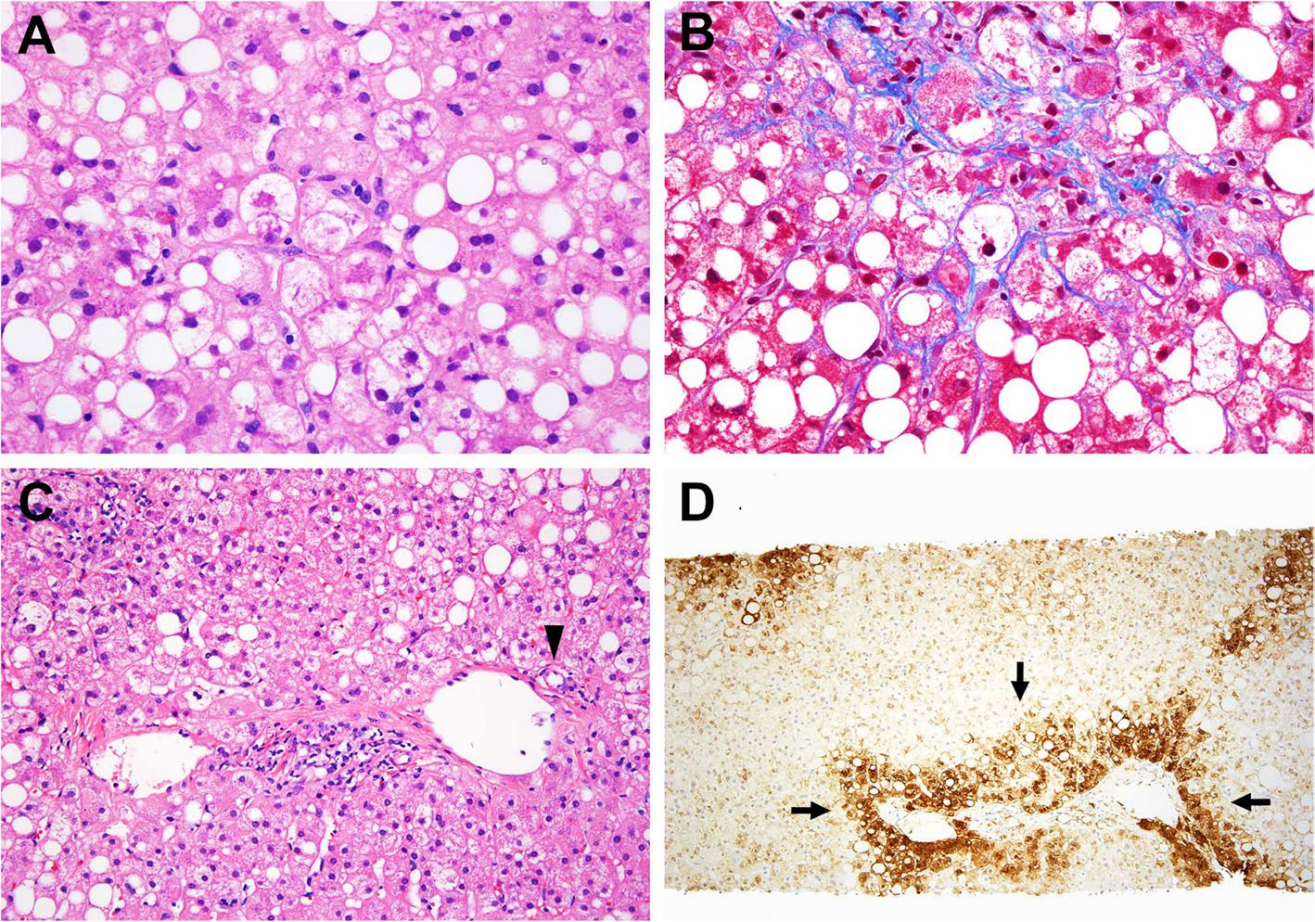
Pathologic features of non-alcoholic steatohepatitis (NASH). **A** A constellation of steatosis, ballooned hepatocytes, and lobular inflammation. **B** Centrizonal sinusoidal fibrosis is present around the ballooned hepatocytes. **C** Examples of a centrizonal artery (arrowhead) in a central zone. Glutamine synthetase immunostain is available in this case and highlights pericentral hepatocytes (Arrows)

Pathologic features of steatosis grade, lobular inflammation and glycogenosis were similar between the two groups (Fig. 2AB). The vast majority of cases showed centrizonal distribution of fat with panlobular steatosis identified in 2 of 37 (5%) patients with de novo NASH and none of the cases of recurrent NASH.

Glycogenosis was variably seen in cases of de novo (*n* = 16, 43%) and recurrent NASH (*n* = 5, 26%) at diagnostic biopsy (*p* = 0.2559). More than focal glycogenosis was also seen in a similar proportion of both de novo (*n* = 9, 5%) and recurrent NASH (*n* = 2, 10%). Centrizonal arterialization was identified in a small subset of de novo NASH (5%, *n* = 2) but not in cases of recurrent NASH (Fig. 2CD) Many of the biopsies in both recurrent (42.1%, 8/19) and de novo NASH (56.8%, 21/37, *p* = 0.3991) showed mild lymphocytic lobular inflammation. Moderate lobular inflammation was only rarely seen (5.4%, 2/37) in cases of de novo NASH, and none of the cases showed severe lobular inflammation. There is no difference in the presence of advanced fibrosis (10.5% versus 21.6%, *p* = 0.4668), pericellular fibrosis (47.4% versus 37.8%, *p* = 0.5717) or portal fibrosis (47.4% versus 56.8%, *P* = 0.578) on diagnostic biopsies from recurrent versus de novo NASH.

**Fig. 3:**
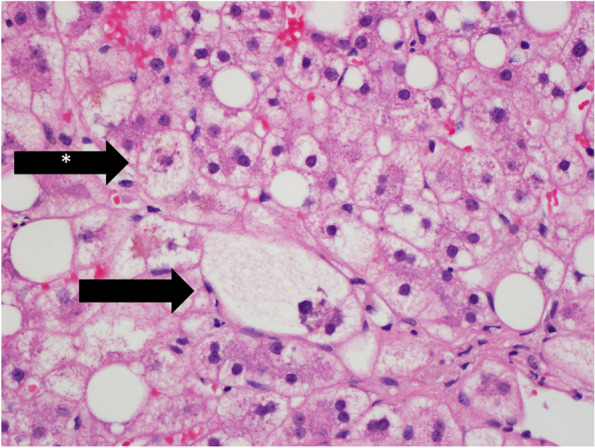
De novo Non-alcoholic steatohepatitis (NASH) with both large ballooned hepatocyte (black arrow) and small ballooned hepatocytes (black arrow with star)

**Fig. 4:**
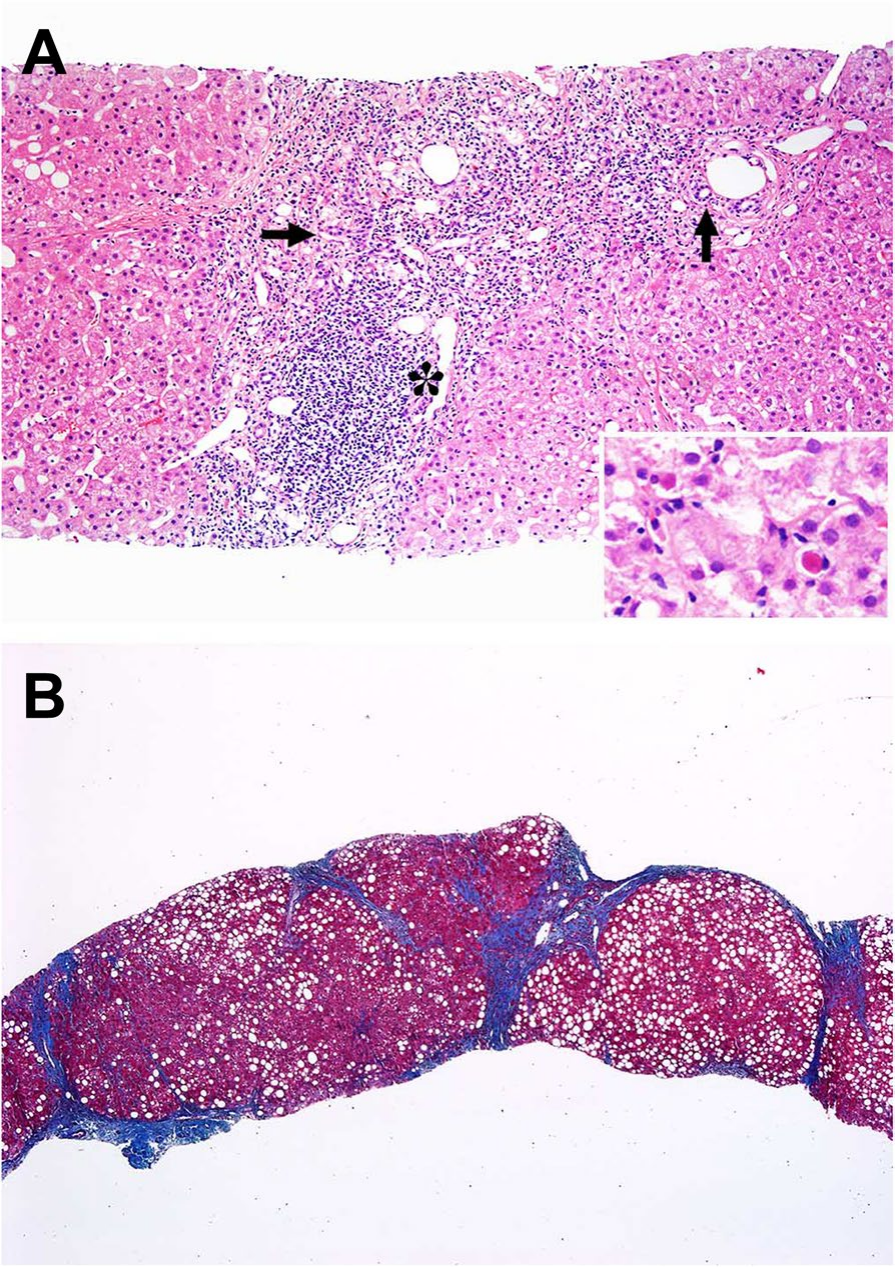
**A** The portal tract shows nodular lymphoid aggregate (asterisk) in this case of recurrent Hepatitis C with more than mild lymphocytic inflammation. The bile ducts (arrows) are intact, H&E stain (inset) shows lobular necroinflammatory activity. **B** A case with cirrhosis, trichrome stain

While the overall presence of any type of ballooned hepatocyte was not different between the two groups (84.2% in recurrent NASH versus 81.1% in de novo NASH, *p* = 1.000), significantly more recurrent NASH biopsies had small ballooned hepatocytes (62.5% of cases) compared to de novo NASH (26.7%) (*p* = 0.03), in which the diagnosis was more often based on large ballooned hepatocytes (see Fig. 3). Recurrent NASH biopsies were also less likely to show “more than mild” portal inflammation (5% vs 40.5%, *p* = 0.0049), although spotty necrosis was frequently seen in diagnostic biopsies of both recurrent (*n* = 8/19, 42.1%) and de novo NASH (*n* = 20/37, 54.1%, *P* = 0.5731) (Fig. 4A). De novo NASH is more likely to show “more than mild” portal inflammation even in exclusion of genotype 3 HCV cases (5% in recurrent NASH compared to 36.7% de novo NASH excluding genotype 3 cases, *p* = 0.0167).

Only a small number of de novo NASH patients (*n* = 6) were transplanted for etiologies other than viral hepatitis (i.e., Hepatitis C and Hepatitis B). Of this group, significantly more cases showed more than mild portal inflammation (50% in non-viral de novo NASH compared to 5% in recurrent NASH, *p* = 0.0312). In this cohort of non-viral de novo NASH, 50% (3 of 6) showed ballooned hepatocytes, all of which were larger ballooned hepatocytes. This trend mirrors the findings of the larger group although did not quite meet statistical significance (52.6% small ballooned hepatocytes in patients with recurrent NASH compared to 0% small ballooned hepatocytes in non-viral de novo NASH, *p* = 0.0508).

Lastly, follow-up biopsies were reviewed for the development of fibrosis after the initial diagnosis of steatohepatitis. Patients with de novo NASH were more likely to develop advanced fibrosis (equal to or greater than stage 3) than patients with recurrent NASH (42% of de novo patients compared to 10% of recurrent NASH patients, *p* = 0.006) (Fig. 4B). There was no difference in development of advanced fibrosis in genotype 3 HCV patients compared to other cases of de novo NASH (*p* = 0.128, Log-rank test).

## Discussion

Overall, the clinicopathologic features of de novo and recurrent NASH were relatively similar. There were no significant differences in immunosuppressant medication between the two groups. Patients with recurrent NASH were more likely to have a pre-transplant diagnosis of hyperlipidemia and diabetes, supportive of pre-existing underlying metabolic disease in these patients. The clinical effect of physical activity and direct measurements of insulin resistance were not available in this retrospective study.

In our study, 78% of de novo NASH patients were initially transplanted for hepatitis C virus infection. The association of hepatitis C with metabolic dysfunction and steatosis has been well-established. In the transplant setting, recurrent HCV is a risk factor for the development of de novo post-transplant diabetes mellitus [26]. Studies have shown that HCV core proteins can activate SREBP1 and 2, inhibiting microsomal triglyceride transfer protein (MTP) activity and impairing peroxisome proliferator-activated receptor (PPAR) expression, thereby directly impacting lipid metabolism and causing hepatic fat accumulation [27]. HCV genotype 3 has been shown to be associated with high grade steatosis, and to have direct viropathic effects on development of hepatic steatosis [27–29]**.** The HCV virus has also been shown to contribute to hepatic steatosis through the induction of oxidative stress [29]. Many of the cases of recurrent NASH in our study showed less severe histology, including less severe portal inflammation and smaller ballooned hepatocytes. Our distinction between large and small ballooned hepatocytes is similar to recent designations as “classic” and “non-classic” ballooned hepatocytes, respectively [30, 31]. Expert hepatopathologists recognize a range in ballooned hepatocyte cell size, including smaller ballooned cells, in establishing a diagnosis of NASH [32].

Given the large percentage of patients with de novo NASH with underlying HCV, a confounding effect of the virus and/or antiviral treatments cannot be entirely excluded; however, similar findings were seen in our small cohort of de novo NASH patients transplanted for etiologies other than viral hepatitis. This less severe histology in recurrent NASH may represent detection of an earlier phase of non-alcoholic steatohepatitis or possibly may represent the effect of prior or ongoing effect of targeted therapy. Therapeutic modalities such as Vitamin E and Pioglitazone can decrease the severity of NASH histology, including decreased portal inflammation and number of ballooned hepatocytes, although interestingly, histologic improvement after therapeutic intervention may not correlate with resolution of NASH [33]. Further studies are necessary to evaluate whether specific therapeutic interventions in the post-transplant setting may contribute to these histologic differences.

The diagnosis of recurrent NASH was made significantly sooner after transplantation than the diagnosis of de novo NASH. Despite milder histology in these cases, 10% of patients with recurrent steatohepatitis in this study developed severe fibrosis. Therefore, these findings have diagnostic significance and argue for careful review for smaller ballooned hepatocytes in cases of recurrent NASH. It is possible that this represents a more rapid onset of disease, but we cannot exclude that this finding represents differences in post-transplant surveillance, effect of prior therapy or other confounding factors, and further studies are necessary. Given the retrospective nature of this study, pre-transplant biopsies are unavailable in the majority of the recurrent NASH patients to compare morphologic features. Further prospective studies are necessary to compare the clinicopathologic features of recurrent NASH pre- and post-transplant.

In a prior study published by Vallin et al., bridging fibrosis occurred at 5 years in 71% of patients with recurrent NAFLD vs. 12.5% of patients with de novo NAFLD, suggesting that recurrent NAFLD is a more severe disease with an earlier onset than de novo NAFLD [34]. In our study, 86% (*n* = 25) of patients transplanted for hepatitis C cirrhosis showed histologic evidence of recurrent hepatitis C prior to, or concurrent with, a diagnosis of de novo NASH, and 70% of the cases transplanted for HCV [*n* = 20] had evidence of post-transplant hepatitis C on viral load testing. Given the high prevalence of HCV infection in the de novo NASH population we cannot make conclusions about the independent impact of NASH on fibrosis progression in this study, but ongoing study of the growing number of patients transplanted for NASH alone will allow for this distinction.

Lastly, centrizonal arterialization was identified in only a minor subset of cases of post-transplant NASH. Nonetheless, it is important to recognize the presence of centrizonal arteries in post-transplant biopsies to avoid misdiagnosis of a post-transplant case as ductopenia in the transplant setting.

## Conclusions

Overall, our findings indicate distinct clinicopathologic features for de novo NASH and recurrent NASH. Cases of recurrent NASH in our study showed less severe histology, including less severe portal inflammation and smaller ballooned hepatocytes, and the diagnosis of recurrent NASH was made significantly sooner after transplantation than the diagnosis of de novo NASH.

The mechanism of disease for these two scenarios is unknown, but given that the autonomic nervous system has an important role in regulation of hepatic homeostasis and lipid metabolism, as well as in the pathogenesis of metabolic syndrome [35, 36], there is no reason to expect that post-transplant NASH (in which the allograft is denervated) will follow the same natural history as conventional NASH and further study is warranted.

## Data Availability

The datasets used and/or analyzed during the current study are available from the corresponding author on reasonable request.
